# Supplementary material for: Expanding the Clinical Spectrum of CEP290 Variants: A Case Report on Non-Syndromic Retinal Dystrophy with a Mild Phenotype
Source: Genes (Basel). 2024 Dec 9;15(12):1584. doi: 10.3390/genes15121584 (PMC11675463; doi:10.3390/genes15121584)
Supplement: Supplementary file 1 [file genes-15-01584-s001.zip › genes-3308832-supplementary.pdf]

Supplementary Material

1 Supplementary Data

The list of 489 genes analyzed through whole exome sequencing (WES) in both siblings is as follows: *ABCA4, ABCC6, ABHD12, ACBD5, ACO2, ACOX1, ADAM9, ADAMTS18, ADAMTS9, ADGRA3, ADGRV1, ADIPOR1, AFG3L2, AGBL5, AHI1, AHR, AIPL1, ALG6, ALMS1, ALPK1, ANKS6, ANTXR1, AP3B1, AP3D1, APOB, APOE, ARHGAP31, ARHGEF18, ARL13B, ARL2, ARL2BP, ARL3, ARL6, ARMC9, ARMS2, ARSG, ARV1, ASRGL1, ATAD3A, ATF6, ATOH7, ATP1A3, ATXN2, ATXN7, B9D1, B9D2, BBIP1, BBS1, BBS10, BBS12, BBS2, BBS4, BBS5, BBS7, BBS9, BEST1, BLOC1S3, BLOC1S5, BLOC1S6, BMP4, BTD, C12orf65, C19orf12, C1QTNF5, C2, C2CD3, C3, C8orf37, C9, CA4, CABP4, CACNA1F, CACNA2D4, CAPN5, CASK, CC2D2A, CCDC28B, CCDC51, CCT2, CDH16, CDH23, CDH3, CDHR1, CDK10, CEP104, CEP120, CEP164, CEP19, CEP250, CEP290, CEP41, CEP78, CEP83, CERKL, CFAP20, CFAP410, CFB, CFH, CFHR1, CFHR3, CFI, CHM, CIB2, CISD2, CLCC1, CLDN19, CLEC3B, CLN3, CLN5, CLN6, CLN8, CLRN1, CLUAP1, CNGA1, CNGA3, CNGB1, CNGB3, CNNM4, COL11A1, COL11A2, COL18A1, COL2A1, COL4A3, COL4A4, COL4A5, COL6A6, COL9A1, COL9A2, COL9A3, CPE, CPLANE1, CRB1, CRB2, CRX, CSPP1, CST3, CTC1, CTNNA1, CTNNB1, CTSD, CWC27, CX3CR1, CYP2U1, CYP4V2, DAG1, DCDC2, DCT, DHDDS, DHX38, DLG1, DNAJC17, DNAJC19, DNAJC21, DNM1L, DOCK6, DRAM2, DTHD1, DTNBP1, EDN3, EDNRB, EFEMP1, ELOVL4, EMC1, ENSA, ERCC6, ESPN, EXOSC2, EYS, FAM149B1, FAM161A, FAM57B, FBLN5, FBN3, FDX2, FDXR, FLVCR1, FRMD7, FSCN2, FZD4, GDF6, GDPD1, GLIS2, GNAT1, GNAT2, GNB3, GNPTG, GPR143, GPR179, GRK1, GRM6, GRN, GUCA1A, GUCA1B, GUCY2D, HACE1, HADHA, HARS, HGSNAT, HK1, HKDC1, HMCN1, HMX1, HPS1, HPS3, HPS4, HPS5, HPS6, HTRA1, IDH3A, IDH3B, IFT122, IFT140, IFT172, IFT27, IFT43, IFT52, IFT74, IFT80, IFT81, IMPDH1, IMPG1, IMPG2, INPP5E, INVS, IQCB1, IRX1, ITM2B, JAG1, KCNJ13, KCNV2, KIAA0556, KIAA0586, KIAA0753, KIAA1549, KIF11, KIF14, KIF3B, KIF7, KITLG, KIZ, KLHL7, LAMA1, LAMA5, LAMB2, LARGE2, LCA5, LOXL3, LRAT, LRIT3, LRMDA, LRP2, LRP5, LYST, LZTFL1, MAK, MANBA, MAPKAPK3, MAPKBP1, MC1R, MCAT, MCOLN1, MECR, MERTK, MFN2, MFRP, MFSD8, MITF, MKKS, MKS1, MLPH, MMACHC, MTPAP, MTTP, MVK, MYO5A, MYO7A, NBAS, NDP, NDUFS1, NEK1, NEK2, NEK8, NEUROD1, NGLY1, NMNAT1, NPHP1, NPHP3, NPHP4, NR2E3, NR2F1, NRL, NUMB, NYX, OAT, OCA2, OFD1, OPA1, OPA3, OPN1LW, OPN1MW, OPN1SW, OR2W3, OTX2, P3H2, PANK2, PAX3, PAX6, PCARE, PCDH15, PCYT1A, PDE6A, PDE6B, PDE6C, PDE6D, PDE6G, PDE6H, PDXK, PDZD7, PEX1, PEX10, PEX11B, PEX12, PEX13, PEX14, PEX16, PEX19, PEX2, PEX26, PEX3, PEX5, PEX6, PEX7, PGK1, PHYH, PIBF1, PITPNM3, PLA2G5, PLK4, PMM2, PNPLA6, POC1B, POC5, POGZ, POLG, POLG2, POMGNT1, PPP2R3C, PPT1, PQLC2, PRCD, PRDM13, PROKR2, PROM1, PRPF3, PRPF31, PRPF4, PRPF6, PRPF8, PRPH2, PRPS1, RAB27A, RAB28, RAX2, RB1, RBP3, RBP4, RCBTB1, RD3, RDH11, RDH12, RDH5, REEP6, RGR, RGS9, RGS9BP, RHBDD2, RHO, RIMS1, RIMS2, RLBP1, ROM1, RP1, RP1L1, RP2, RP9, RPE65, RPGR, RPGRIP1, RPGRIP1L, RRM2B, RS1, RTN4IP1, SAG, SAMD11, SCAPER, SCLT1, SDCCAG8, SEMA4A, SEMA6B, SIX6, SLC24A1, SLC24A2, SLC24A5, SLC25A46, SLC37A3, SLC38A8, SLC39A12, SLC45A2, SLC4A3, SLC4A4, SLC4A7, SLC52A2, SLC6A6, SLC7A14, SMARCA4, SNAI2, SNRNP200, SNX10, SOX10, SOX3, SPATA7, SPG7, SPP2, SRD5A3, SSBP1, STX3, SUFU, TCTN1, TCTN2, TCTN3, TEAD1, TGFBR2, TIMM8A, TIMP1, TIMP3, TLR3, TLR4, TMEM107, TMEM126A, TMEM138, TMEM216, TMEM231, TMEM237, TMEM67, TOGARAM1, TOPORS, TPP1, TRAF3IP1, TREX1, TRIM32, TRNT1, TRPM1, TSFM, TSPAN12, TTC21B, TTC8, TTLL5, TTPA, TUB, TUBB3, TUBB4B, TUBGCP4, TUBGCP6, TULP1, TXNDC15, TYR, TYRP1, UCHL1, UNC119, USH1C, USH1G, USH2A, USP45, VCAN, VHL, VPS13B, WDPCP, WDR19, WDR34, WFS1, WHRN, XPNPEP3, YARS, YME1L1, YPEL2, ZNF408, ZNF423, ZNF513, ZNHIT3*.

2 Supplementary Figures

1. Dark-adapted 0.01 ERG Stimulator: Ganzfeld

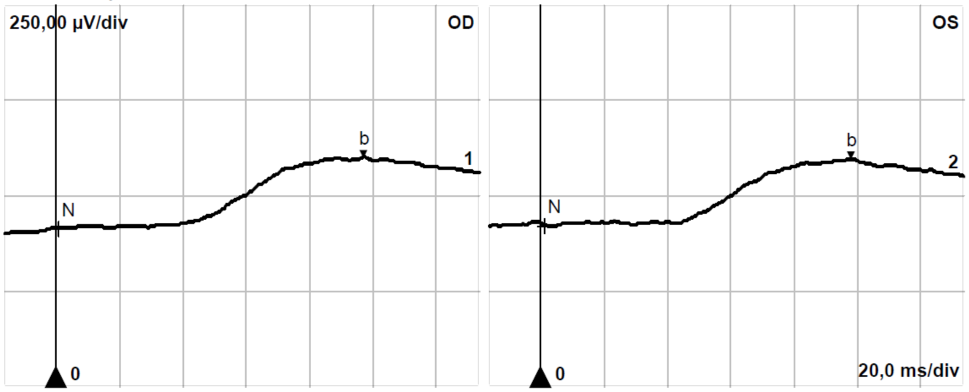

Etapa	b [ms]	b-wave [µV]	Avgs.
Normal	67,0-91,0	118,0-225,0	
1: OD-1	97,3 (!)	190,4	5
2: OS-2	98,1 (!)	176,6	5

2. Dark-adapted 3.0 ERG Stimulator: Ganzfeld

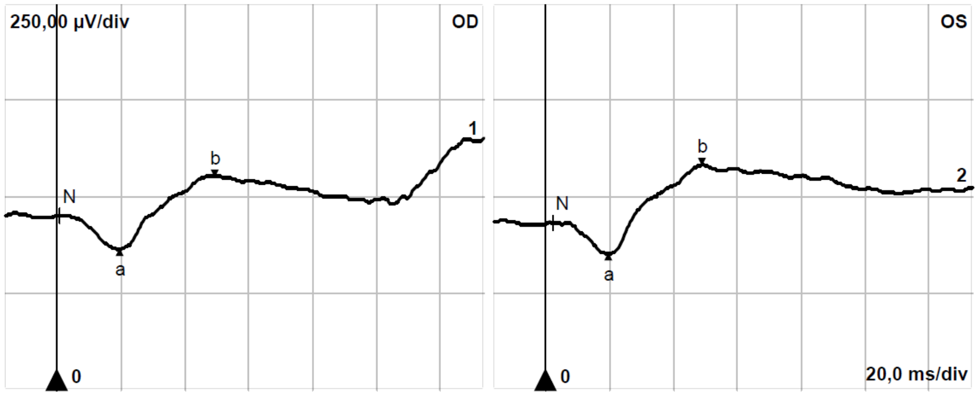

Etapa	a [ms]	b [ms]	a-wave [µV]	b-wave [µV]	b/a	Avgs.
Normal	15,9-22,0	35,5-51,4	63,0-272,0	82,0-380,0	1,5-2,6	
1: OD-1	19,6	49,8	88,0	192,4	2,2	5
2: OS-2	19,9	49,2	79,9	233,5	2,9 (!)	5

3. Dark-adapted 3.0 OPs Stimulator: Ganzfeld

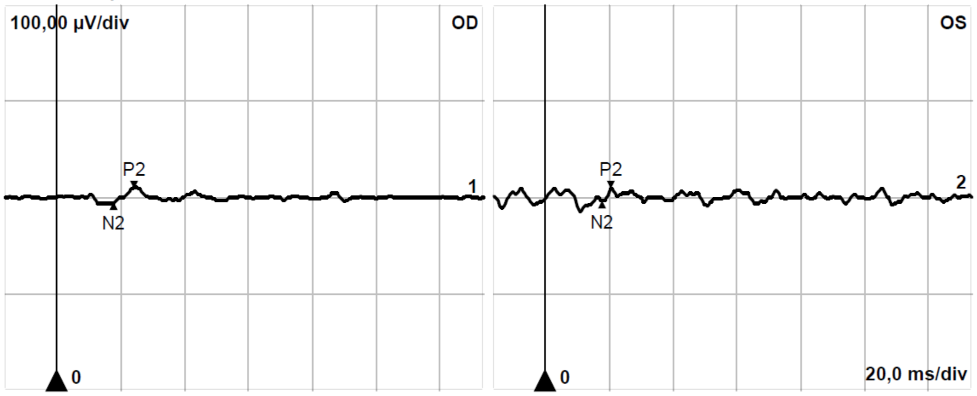

Etapa	P2 [ms]	OS2 [µV]	Avgs.
Normal	23,2-27,6	4,6-51,2	
1: OD-1	24,3	17,6	3
2: OS-2	20,8 (!)	14,9	3

4. Dark-adapted 10.0 ERG Stimulator: Ganzfeld

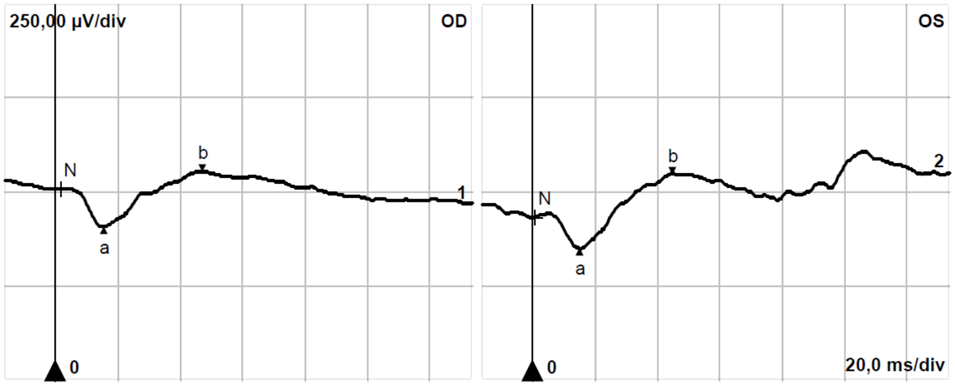

Etapa	a [ms]	b [ms]	a-wave [µV]	b-wave [µV]	b/a	Avgs.
Normal	14,7-16,4	36,4-49,9	110,0-317,0	127,0-420,0		
1: OD-1	15,5	47,5	103,2 (!)	150,6	1,5	5
2: OS-2	15,2	44,8	85,9 (!)	203,6	2,4	5

5. Light-adapted 3.0 ERG Stimulator: Ganzfeld

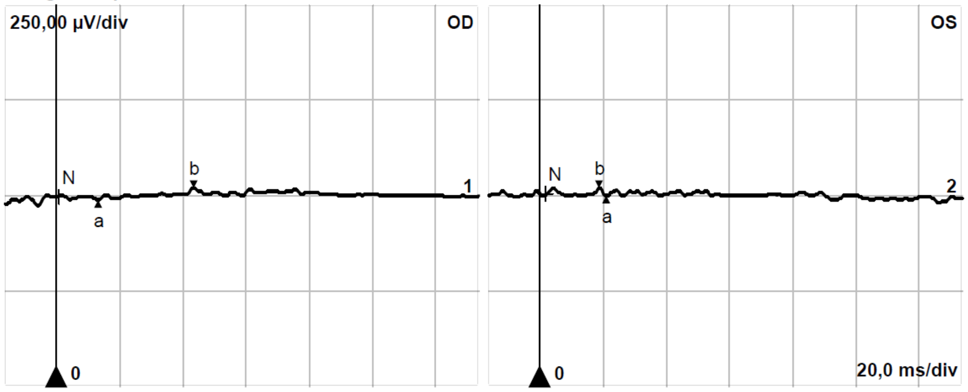

Etapa	a [ms]	b [ms]	a-wave [µV]	b-wave [µV]	Avgs.
Normal	12,9-16,7	29,6-33,5	10,4-55,0	39,6-184,0	
1: OD-1	13,5	43,7 (!)	11,0	36,5 (!)	5
2: OS-2	21,1 (!)	19,0 (!)	6,1 (!)	25,9 (!)	5

6. Light-adapted 3.0 Flicker 30 Hz Stimulator: Ganzfeld

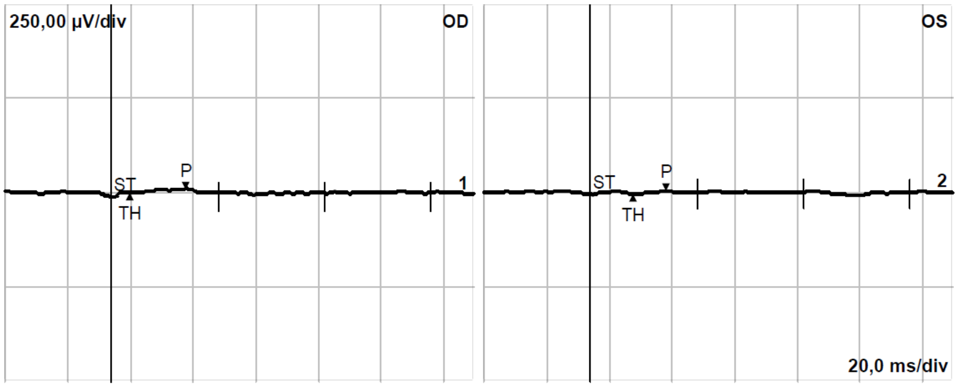

Etapa	IT-b [ms]	A-b [µV]	Avgs.
Normal	26,0-30,0	34,4-173,0	
1: OD-1	24,0 (!)	13,7 (!)	12
2: OS-2	24,6 (!)	13,1 (!)	12

Figure S1: ffERG results for proband 2. Panels (1-4) represent the scotopic ERG responses, while panels (5-6) illustrate the photopic ERG responses.

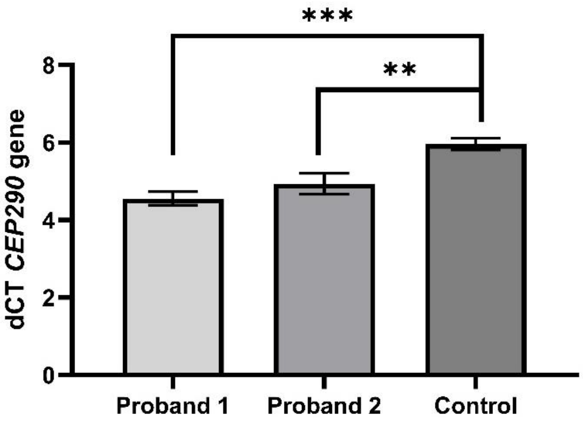

Figure S2: qPCR analysis of *CEP290* gene expression. The graph illustrates the relative expression levels of the *CEP290* gene in probands 1 and 2 compared to a control sample, normalized to *GAPDH* expression. A reduction in CEP290 expression was observed in proband 1 ($p = 0.0004$) and proband 2 ($p = 0.004$). **: $p \leq 0.01$; ***: $p \leq 0.001$.

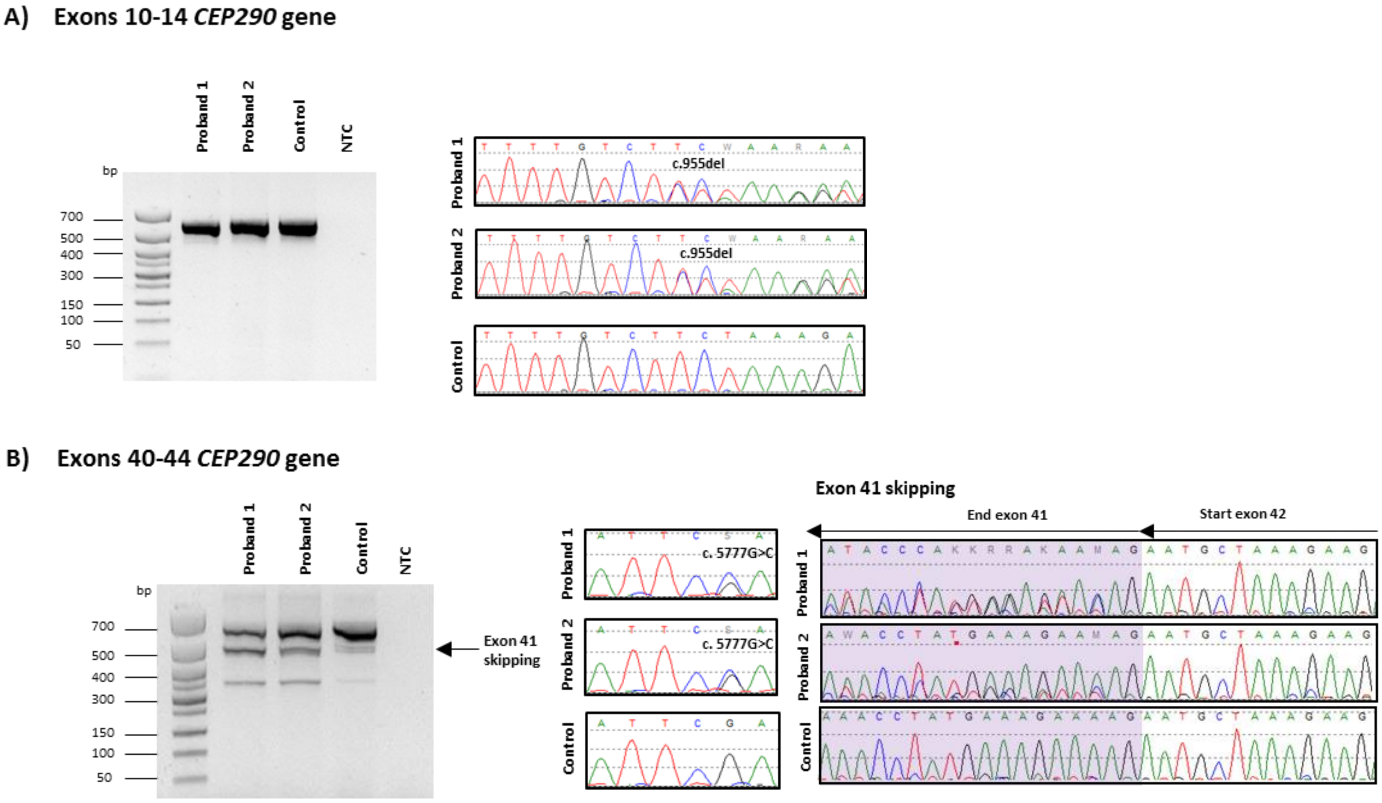

Figure S3: Impact of identified *CEP290* variants on splicing. A) Amplification of exons 10-14 of the *CEP290* gene shows that the c.955del variant does not affect splicing. **B)** Amplification of exons 40-44 reveals no skipping of exon 42, which contains the c.5777G>C variant. However, exon 41 skipping is observed. The agarose gel demonstrates that the band corresponding to exon 41 skipping is more prominent in the probands compared to the control sample. NTC, no template control.

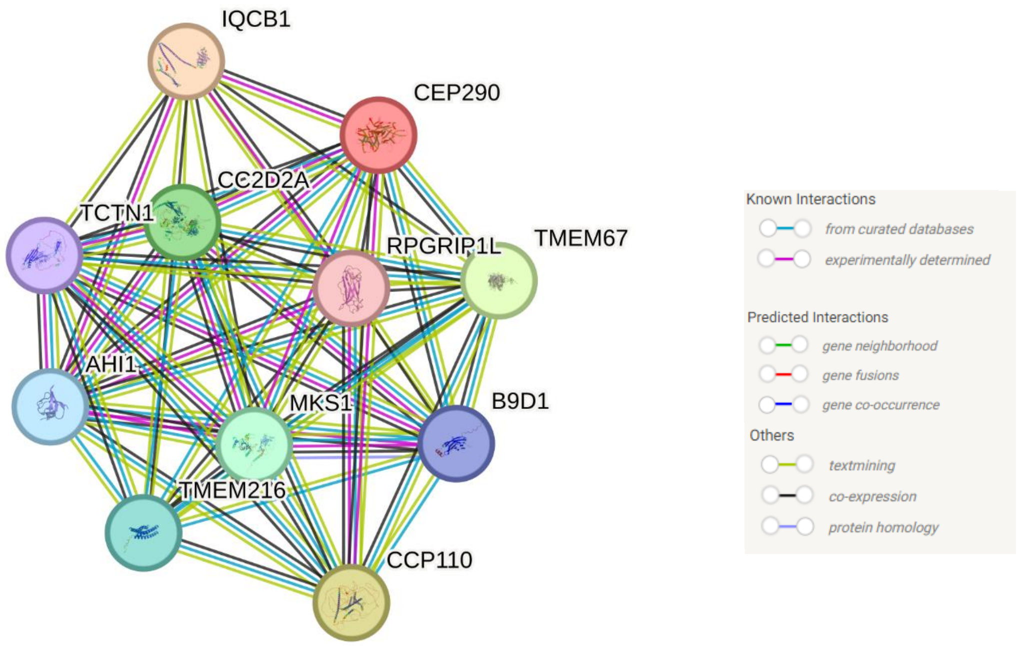

Figure S4: Protein-protein interaction network of CEP290. This figure illustrates the protein-protein interaction network of CEP290, including key interactors such as AHI1, CC2D2A, and RPGRIP1L. These proteins are well-established binding partners of CEP290 and have been implicated in modulating the phenotype of *CEP290*-related disorders.

3 Supplementary Tables

Table S1. Primers used to evaluate the effect on splicing of the variants identified.

Primer name	Primer sequence (5′-3′)	Amplicon length
CEP290 10-14	TGTAAAACGACGGCCAGT **GAATGAAAGCTATTGTGCATC**<br>CAGGAAACAGCTATGACC **GTTCAGCTGTTCTCTCAGC**	546 pb
CEP290 40-44	TGTAAAACGACGGCCAGT **GATCAAGAGAATGATGAACTG**<br>CAGGAAACAGCTATGACC **CTTCTACAACAGAATCTCGAG**	556 pb

The specific sequence is highlighted in bold, while the unmarked sequence corresponds to M13 forward and reverse, respectively.

Table S2. Variants in modifier genes (*AHI1, ARL13B, CC2D2A* and *RPGRIP1L*) identified in proband 1.

Proband 1								
**Gene**	**Nucleotide change**	**Aminoacid change**	**Variant type**	**Zygosity**	**dbSNP**	**gnomAD**	**Revel**	**SpliceAI**
ARL13B	c.1043C>G	p.(Thr348Ser)	missense	HOM_ALT	rs33944211	0.0964	0.067	0.01
CC2D2A	c.247+26A>G	-	intronic	HOM_ALT	rs10000250	0.8602	-	0
CC2D2A	c.1519A>G	p.(Lys507Glu)	missense	HET	rs144439937	0.00638	0,683	0
CC2D2A	c.1764+45T>G	-	intronic	HET	rs1558572	0.7535	-	0
CC2D2A	c.2003+19C>T	-	intronic	HET	rs17476642	0.1093	-	0
CC2D2A	c.2003+730A>G	-	intronic	HET	rs1125378	0.7563	-	0.03
CC2D2A	c.3183-8T>C	-	3'UTR	HOM_ALT	rs13121363	0.7061	-	0
CC2D2A	c.3288+41A>C	-	intronic	HOM_ALT	rs13116304	0.7073	-	0
CC2D2A	c.4065+28A>T	-	3'UTR	HOM_ALT	rs6832789	0.9856	-	0
CC2D2A	c.*21G>C	0.445	3'UTR	HET	rs1134634	0.5772	-	0
CC2D2A	c.*64T>G	0.556	3'UTR	HET	-	0.000002045	-	0
AHI1	c.3426+13G>A	-	3'UTR	HOM_ALT	rs6914831	0.5876	-	0
AHI1	c.3015A>G	p.(Ser1005Ser)	synonymous	HET	rs41287054	0.01752	-	0
AHI1	c.2961+7_2961+21delinsGACTTTTTTAAAGTTTTAAA	-	Splice site	HET	rs786200964	-	-	0
AHI1	c.2961+17C>A	-	intronic	HET	rs201291897	-	-	0
AHI1	c.2961+16G>A	-	intronic	HET	rs199671265	-	-	0
AHI1	c.2961+14A>T	-	intronic	HET	rs200731111	-	-	0
AHI1	c.2961+9A>C	-	intronic	HET	rs201870233	-	-	0
AHI1	c.2961+8T>A	-	Splice site	HET	rs199887601	-	-	0
AHI1	c.2961+7T>G	-	Splice site	HET	rs200658320	-	-	0
AHI1	c.1780-14C>T	-	intronic	HOM_ALT	rs2757645	0.9044	-	0
RPGRIP1L	c.*55T>A	-	3'UTR	HET	rs4784319	0.3727	-	0.02
RPGRIP1L	c.2959-32G>A	-	intronic	HET	rs7203525	0.3441	-	0.12

*MANE transcripts NM_001174150.2 (*ARL13B*), NM_001378615.1 (*CC2D2A*), NM_001134831.2 (*AHI1*).

Supplementary Material

Table S3. Variants in modifier genes (*AHI1, ARL13B, CC2D2A* and *RPGRIP1L*) in proband 2.

Proband 2								
**Gene**	**Nucleotide change**	**Aminoacid change**	**Variant type**	**Zygosity**	**dbSNP**	**gnomAD**	**Revel**	**SpliceAI**
ARL13B	c.1043C>G	p.(Thr348Ser)	missense	HET	rs33944211	0.0964	0.067	0.01
CC2D2A	c.247+26A>G	-	intronic	HET	rs10000250	0.8602	-	0
CC2D2A	c.1765-24A>G	-	intronic	HET	rs1861044	0.5457	-	0
CC2D2A	c.1764+45T>G	-	intronic	HOM_ALT	rs1558572	0.7535	-	0
CC2D2A	c.2003+19C>T	-	intronic	HET	rs17476642	0.1093	-	0
CC2D2A	c.2003+730A>G	-	intronic	HOM_ALT	rs1125378	0.7563	-	0.03
CC2D2A	c.3183-8T>C	-	3'UTR	HOM_ALT	rs13121363	0.7061	-	0
CC2D2A	c.3288+41A>C	-	intronic	HOM_ALT	rs13116304	0.7073	-	0
CC2D2A	c.4065+28A>T	-	3'UTR	HOM_ALT	rs6832789	0.9856	-	0
CC2D2A	c.*21G>C	0.445	3'UTR	HET	rs1134634	0.5772	-	0
CC2D2A	c.*64T>G	0.556	3'UTR	HET	-	0.000002045	-	0
AHI1	c.3426+13G>A	-	3'UTR	HET	rs6914831	0.5876	-	0
AHI1	c.3015A>G	p.(Ser1005Ser)	synonymous	HET	rs41287054	0.01752	-	0
AHI1	c.1780-14C>T	-	intronic	HOM_ALT	rs2757645	0.9044	-	0
RPGRIP1L	c.*55T>A	-	3'UTR	HET	rs4784319	0.3727	-	0.02
RPGRIP1L	c.2959-32G>A	-	intronic	HET	rs7203525	0.3441	-	0.12

*MANE transcripts NM_001174150.2 (*ARL13B*), NM_001378615.1 (*CC2D2A*), NM_001134831.2 (*AHI1*).

Table S4: Clinical and genetic characteristics of individuals with biallelic pathogenic *CEP290* variants identified in our internal IRD cohort.

Case	Sex	AO	Age	FamHx	Clinical diagnosis	Alleles	Location
**1**	F	1	28	No	Joubert syndrome	c.5227-13G>A, p.(?)<br>c.6271-8T>G, p.(?)	Intron 38<br>Intron 45
**2**	M	1	20	No	Leber Congenital Amaurosis	c.2991+1655A>G, p.(?)<br>c.2991+1655A>G, p.(?)	Intron 26<br>Intron 26
**3**	M	1	14	No	Leber Congenital Amaurosis	c.2991+1655A>G, p.(?)<br>c.2991+1655A>G, p.(?)	Intron 26<br>Intron 26
**4**	M	1	10	No	Leber Congenital Amaurosis	c.2991+1655A>G, p.(?)<br>c.3400_3401del, p.(Asp1134Ter)	Intron 26<br>Exon 29
**5**	F	14	28	No	Retinitis Pigmentosa	c.5932C>T, p.(Arg1978Ter)<br>c.7341dup, p.(Leu2448ThrfsTer8)	Exon 43<br>Exon 54
**6**	M	1	4	No	Leber Congenital Amaurosis	c.41A>G, p.(Asp14Gly)<br>c.133_136del, p.(Gln45LysfsTer3)	Exon 2<br>Exon 3
**7**	M	1	5	No	Leber Congenital Amaurosis	c.2991+1655A>G, p.(?)<br>c.2991+1655A>G, p.(?)	Intron 26<br>Intron 26
**8**	F	10	32	No	Senior-Loken syndrome	c.4813-1G>A, p.(?)<br>c.5736G>A, p.(Trp1912Ter)	Intron 36<br>Exon 42
**9**	M	1	6	No	Leber Congenital Amaurosis	c.2991+1655A>G, p.(?)<br>c.4661_4663del, p.(Glu1554del)	Intron 26<br>Exon 35

F: female, M: male, AO: Age of onset, FamHx: Family history
